# Involvement of dysregulated hippocampal histone H3K9 methylation at the promoter of the BDNF gene in impaired memory extinction

**DOI:** 10.1007/s00213-024-06640-7

**Published:** 2024-06-28

**Authors:** Kenichi Oga, Manabu Fuchikami, Hironori Kobayashi, Tatsuhiro Miyagi, Sho Fujita, Satoshi Fujita, Satoshi Okada, Shigeru Morinobu

**Affiliations:** 1 Department of Psychiatry and Neuroscience, Division of Graduate School of Biomedical and Health Sciences, Hiroshima University, Minami-Ku, Kasumi 1-2-3, Hiroshima City, Hiroshima Japan; 2 Department of Psychology, School of Faculty of Health and Wellness Sciences, Hiroshima International University, Kure, Japan

**Keywords:** Extinction of fear memory, Post-traumatic stress disorder, Histone H3K9 methylation, Brain-derived neurotrophic factor, Hippocampus

## Abstract

**Rationale:**

Since the precise mechanisms of posttraumatic stress disorder (PTSD) remain unknown, effective treatment interventions have not yet been established. Impaired extinction of fear memory (EFM) is one of the core symptoms of PTSD and is associated with stress-induced epigenetic change in gene expression.

**Objectives:**

In this study, we examined whether the involvement of histone H3 lysine 9 dimethylation (H3K9me2) in EFM is mediated through brain-derived neurotrophic factor (BDNF) expression in the hippocampus, and whether BIX01294, a selective G9a and GLP histone methyltransferase inhibitor, could be treatment for impaired EFM in an animal model of PTSD.

**Methods:**

The single prolonged stress (SPS) paradigm was used to model PTSD. We measured BDNF mRNA levels by RT-PCR, and H3K9me2 levels in the BDNF gene promoters by chromatin immunoprecipitation-qPCR. After undergoing contextual fear conditioning and hippocampal injection of BIX01294, male rats were subjected to extinction training and extinction testing and their freezing times and BDNF mRNA levels were measured.

**Results:**

Compared to sham rats, SPS rats showed decreased BDNF mRNA levels 2 h after extinction training, no significant changes in levels of global H3K9me2 prior to extinction training, and increased levels of H3K9me2 in BDNF gene promoter IV, but not in BDNF gene promoter I. Administration of BIX01294 ameliorated the decrease in BDNF mRNA levels 2 h after extinction training and subsequently alleviated impaired EFM in extinction tests in SPS rats.

**Conclusion:**

We conclude that reduced hippocampal levels of BDNF mRNA due to increase in H3K9me2 levels may play a role in PTSD-associated EFM impairment, and BIX01294 could be a PTSD treatment option.

**Supplementary Information:**

The online version contains supplementary material available at 10.1007/s00213-024-06640-7.

## Introduction

Persistent intrusive symptoms associated with traumatic events, such as flashbacks, undesired confounding memories, and nightmares are reported to be diagnostic criteria for posttraumatic stress disorder (PTSD) (Lawrence-Wood et al. 2016; Messent 2013). Impaired extinction of fear memory (EFM) has been proposed to be closely involved in the development of intrusive symptoms. In EFM, the conditioned fear response is reduced after repeated exposure to a conditioned stimulus in the absence of a noxious unconditioned stimulus (Miedl et al. 2020; Milad et al. 2009; Rothbaum and Davis 2003). In fact, impaired EFM was demonstrated in patients with PTSD by fear conditioning experiments(Blechert et al. 2007), and a poor capacity for extinction learning was reported to be a risk factor of PTSD in firefighters (Guthrie and Bryant 2006). In addition, EFM is suggested to play a pivotal role in efficacious trauma-focused psychotherapy for PTSD. For example, in the prolonged exposure therapy process, it is postulated that confrontation with frightening yet safe stimuli that are compatible with preexisting fear memories must be assimilated into a new memory (Foa and Kozak 1986). In this context, EFM is the new learning of a safe situation rather than an elimination of a fear memory.

Although it is not fully known what role brain regions play in fear memory, fear memory mechanisms have been reported to be closely associated with the amygdala, prefrontal cortex (PFC), and hippocampus (Sotres-Bayon et al. 2004, 2006). Regarding EFM, the role of the ventral hippocampus working in concert with the infralimbic cortex (ILC) is reported to be important (Burgos-Robles et al. 2009; Sierra-Mercado et al. 2011; Sotres-Bayon and Quirk 2010). Regarding molecular mechanisms of successful EFM in these brain regions, a series of studies using the micro-infusion of brain-derived neurotrophic factor (BDNF) and its antibody suggested that elevated BDNF levels in the ventral hippocampus and subsequent increase in BDNF levels in the ILC or direct transport of BDNF into the ILC were important (Peters et al. 2010; Rosas-Vidal et al. 2014). Recently, we have reported that in the single prolonged stress (SPS) paradigm, a rodent model mimicking numerous pathophysiological and behavioral characteristics of PTSD (Keller et al. 2015; Knox et al. 2012; Souza et al. 2017; Yamamoto et al. 2009), impaired EFM is induced by decreasing BDNF in the medial prefrontal cortex (mPFC) and hippocampus, and by decreasing phosphorylation of TrkB in the ILC (Kataoka et al. 2019). In addition, clinical findings demonstrating the association of BDNF dysregulation with the pathophysiology of PTSD (Green et al. 2013; Zhang et al. 2014, 2016) further support the potency of BDNF as a novel therapy for PTSD.

On the other hand, recent studies investigating the mechanisms of long-term memory formation have suggested the involvement of epigenetic machinery regulating the gene transcription essential to the development of persistent memory (Kwapis and Wood 2014; Lattal and Wood 2013). Epigenetic mechanisms, such as histone acetylation (Barrett and Wood 2008), histone phosphorylation (Kouzarides 2007), histone methylation (Jarome and Lubin 2013), DNA methylation (Zovkic and Sweatt 2013), and nucleosome remodeling (Vogel-Ciernia and Wood 2014), have been implicated in the formation of long-term memory. Especially, histone acetylation is one of the most investigated epigenetic mechanisms associated with EFM. A series of studies revealed that contextual fear conditioning (CFC) induces histone H3 acetylation in the hippocampus and that histone deacetylase (HDAC) inhibitors (Levenson et al. 2004) enhance long-term fear memory and EFM (Bredy and Barad 2008; Gräff et al. 2014; Lattal et al. 2007; Marek et al. 2011; Stafford et al. 2012). Also, we have previously reported that administration of an HDAC inhibitor immediately after extinction training ameliorated the impaired EFM fear in SPS rats, and this effect was associated with an increase in histone acetylation and thereby enhancement of NR2B and CaMKII in the hippocampus (Matsumoto et al. 2013). In addition, increased binding of acetylated histone to the promoter region of the BDNF gene and subsequent HDAC inhibitor-induced increase in BDNF levels were reported to enhance EFM (Bredy et al. 2007; Fujita et al. 2012). Likewise, it has been reported that dimethylation of histone H3 lysine 9 (H3K9me2), which is regulated by histone methyltransferase (HMT) G9a (Shinkai and Tachibana 2011), is also associated with the formation and consolidation of fear memory and EFM in the rat hippocampus and amygdala. Most recently, Zhao and his colleagues found that traumatic stress experiences induce PTSD-like behaviors through increase in H3K9me2 levels and the repression of BDNF transcription in the hippocampus, PFC, and amygdala (Zhao et al. 2020, 2022). Moreover, HMT G9a inhibitors could improve traumatic stress-induced PTSD-like behaviors and ameliorate the increase in H3K9me2 levels (Gupta-Agarwal et al. 2012, 2014). This accumulating evidence suggests that measurement of BDNF mRNA levels in a rat PTSD model could be used to elucidate the involvement of H3K9me2 in impaired EFM and to examine the effect of HMT G9a inhibitors on EFM impairment and its association with BDNF mRNA level.

Although the ILC is shown to be involved in the development of EFM, BDNF in the ILC transported from the hippocampus, is reported to be crucial in the development of EFM in rodents (Peters et al. 2010; Rosas-Vidal et al. 2014). In this context, we focused on the expression of BDNF and levels of H3K9me2 in the ventral hippocampus in the present work. We firstly examined BDNF mRNA levels in the hippocampus and in the cerebellum (as a control region) of SPS rats before CFC, and before and after extinction training 24 h after CFC. Next, we evaluated global levels of H3K9me2 in the hippocampus and in the cerebellum (as a control region) of SPS rats by western blotting and levels of H3K9me2 in the promoter regions of the BDNF gene by chromatin immunoprecipitation (ChIP)-qPCR in the hippocampus of SPS rats. Lastly, we examined whether hippocampal infusion of BIX01294, an HMT G9a inhibitor, restored depressed levels of BDNF mRNA and relieved EFM impairment.

## Materials and methods

**Fig. 1:**
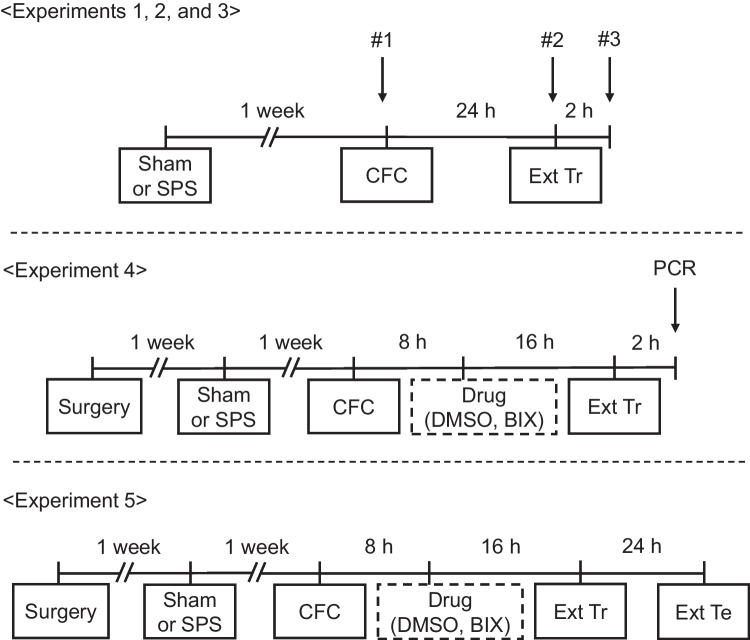
Experimental procedures. CFC, contextual fear conditioning; Ext Tr, extinction training; Ext Te, extinction test; SPS, single prolonged stress; DMSO, dimethyl sulfoxide; BIX, BIX01294, HMT G9a inhibitor

The timelines of all experiments are depicted in Fig. 1.

### Animals

Adult Sprague–Dawley rats (Jackson Laboratory Japan, Inc., Yokohama, Japan) weighing 300 to 350 g were group housed (2–3 per cage), maintained on a 12-h light/dark cycle, and given ad libitum access to food and water. All experiments took place during the light cycle. Because of the bidirectional (facilitation and suppression) influence of the estrous cycle and gonadal hormone on learned fear (Day and Stevenson 2020), we used only male rats in this study.

All animal experiments followed the Guidelines of the Science Council of Japan for Proper Conduct of Animal Experiments and were approved by the Hiroshima University Institutional Animal Care and Use Committee.

### Single Prolonged Stress (SPS)

SPS was performed in 3 steps as previously described (Araki et al. 2020; Kataoka et al. 2019; Liberzon et al. 1997; Omura et al. 2021): 2 h of restraint, 20 min of forced swimming, and anesthetization to a state of deep anesthesia using ether. In experiments 4 and 5, SPS was undertaken after a week of recovery from stereotaxic surgery. In addition, the rats were left to rest undisturbed for a one-week period after exposure to SPS.

### Contextual fear conditioning and assessment of EFM

To evaluate EFM, extinction training and extinction testing following contextual fear conditioning were performed as previously described (Kataoka et al. 2019; Omura et al. 2021). On the day of contextual fear conditioning, rats were placed into a conditioning context without any stimulation for 180 s in a conditioning container (W500 x D280 x H325 mm), footshocked (4 s, 0.8 mA) through a metal grid placed on the bottom of the container on two occasions separated by a 30-s intertrial interval, and left in the container for an additional 1 min. On the following day, extinction training was done by placing the footshocked rats in the same container without exposure to footshock for 10 min. In experiment 5, extinction recall was tested on following day in a similar manner to assess the effects of BIX01294 on EFM impairment (Fig. 1). In the extinction training and testing sessions, the duration of the freezing behavior was measured, and the percentage of the 10-min experimental period during which the mice exhibited freezing behavior was calculated.

### Stereotaxic surgery

For the studies using drug administration into the hippocampus (Experiments 3,4), stereotaxic surgery was performed 1 week before SPS (Fig. 1).

Rats were anesthetized with a combination of pentobarbital (40 mg/kg, ip) and isoflurane inhalation. Bilateral guide cannulas (22-gauge; Plastics one, Roanoke, VA, USA) were implanted in the hippocampi (-6.3 mm AP; ± 5.0 mm ML; 5.5 mm DV), fixed to stainless-steel screws with light-curing adhesive, and then fixed to the entire skull with acrylic resin. A triple antibacterial agent was applied to the surgical site after surgery and an analgesic agent (carprofen; 5 mg/kg) was given subcutaneously for 3 days.

### Intra hippocampal drug administration

BIX01294 (Adipogen, Füllinsdorf, Switzerland) or DMSO was administered bilaterally into the hippocampus through an injection cannula (28-gauge) inserted into a chronically implanted guide cannula. The reduction of H3K9me2 levels at G9a responsive promoter by BIX01294 treatment was reported to be nearly 50% in 6 h and further declined to less than 20% in 12 h, and less than 10% in 24 h (Kubicek et al. 2007). The time of drug administration (16 h before extinction training) was set to utilize the pharmacokinetic properties of the drug at the time of extinction training. BIX01294 was dissolved in DMSO at 5 mg/ml (10.2 mM), adjusted to 45 μM with saline (a concentration previously used for hippocampal administration of this drug) (Gupta-Agarwal et al. 2012), and injected at a dose of 0.5 μL/side and at a rate of 0.1 μL/min.

In experiment 4, the placements of the injection cannula were verified manually before collecting the hippocampus for the later mRNA extraction. In experiment 5, after the completion of extinction testing, rats were deeply anesthetized with chloral hydrate (250 mg/kg, i.p.) and transcardially perfused with 10% formalin. ice-cold PBS followed by 10% buffered formalin. Following perfusion, brains were cut using a Brain matrix (68717, RWD, Sugar land, TX, USA), and the placements of the injection cannula were verified. All infusion sites in experiment 5 are shown in Fig. 6a. Animals with incorrect cannula placement were not included in the study.

### Measurement of BDNF mRNA levels by real-time RT-PCR

Total RNA was extracted from rat hippocampus and cerebellum using an RNAqueous Total RNA Isolation kit (Thermo Fisher Scientific, MA, USA). The cDNA was synthesized using the QuantiTect Reverse Transcription kit (Qiagen, Venlo, the Netherlands). Levels of BDNF mRNA were measured by quantitative RT-PCR using a CFX96 touch real-time PCR detection system (Bio-Rad, CA, USA). Specific primers and probes were selected from TaqMan gene expression assay inventories (Thermo Fisher Scientific, Rn02531967_s1 for BDNF and Rn01775763_g1 for GAPDH). All samples were assayed in duplicate. A standard curve was constructed from standard samples (rat hippocampus), and the levels of BDNF and GAPDH in unidentified samples were quantified by interpolation from this standard curve. The BDNF data were normalized to GAPDH levels in each sample.

### Measurement of global H3K9me2 levels by western blotting

To measure the global levels of H3K9me2 in the hippocampus and in the cerebellum of rats, total histone was extracted using an EpiQuik Total Histone Extraction Kit (Epigentek, Farmingdale, NY, USA). Total protein concentrations were measured using a Protein Assay BCA Kit (Nacalai Tesque, Kyoto, Japan), and 10 μg of protein was separated by electrophoresis on a NuPAGE 4–12% Bis–Tris Gel (Thermo Fisher Scientific). The proteins were transferred from the gel to a polyvinylidene fluoride membrane (Trans-Blot Turbo Mini 0.2 μm PVDF Membrane, Bio-Rad), blocked with tris buffered saline with Tween 20 (TBST) containing 5% skim milk, and probed overnight with the following two primary antibodies: anti-dimethyl histone H3K9 (catalog #4658, 1:1000; Cell Signaling Technology, MA, USA) and anti-histone H3 (catalog #4499, 1:2000; Cell Signaling Technology). Then, the membrane was washed in TBST and incubated with secondary antibodies (donkey anti-rabbit IgG antibody linked to horseradish peroxidase, NA934, 1:50,000; Cytiva, Tokyo, Japan). The immunoblot signals were detected with the ChemiDoc XRS + system (Bio-Rad) and density of the immunoreactive bands was quantified using Image Lab software (Bio-Rad). The H3K9me2 data were normalized to the Histone H3 level in each sample.

### Chromatin immunoprecipitation (ChIP) assays

To determine the levels of H3K9me2 in the promoter regions of the BDNF gene, ChIP assays were performed using an EpiQuik ChIP kit (Epigentek) as previously described (Araki et al. 2020). Briefly, the hippocampus was cut into 1-mm sized pieces and its proteins were cross-linked by incubating with 1% formaldehyde for 20 min. Then, the cross-linking reaction was stopped by adding glycine to a final concentration of 1.25 M. After washing and centrifugation, the tissue was homogenized 20 times using a Dounce tissue grinder. The tissue suspension was centrifuged for 5 min at 5000 rpm, then the supernatant was treated with lysis solution including protease inhibitors and sonicated 10 times at 30-s intervals using a Bioruptor (Tosho Electric Machines, Tokyo, Japan) at maximal power. The tissue lysate was treated with 300 U of micrococcal nuclease (Thermo Fisher Scientific), and 200 mM EDTA was added to stop the nuclease activity. The chromatin lysate was then diluted with ChIP diluent to 20 μL. Input DNA (5 µL of the diluted supernatant prior to immunoprecipitation) was stored and used later for normalization of the data. The chromatin solution was immunoprecipitated with 2 μg of anti-H3K9me2 antibody for 90 min at room temperature. Tests included positive controls, i.e., samples immunoprecipitated with 1 μg of anti-RNA polymerase and negative controls, i.e., samples immunoprecipitated with non-immune rabbit IgG. The DNA-histone complexes were extracted and eluted with DNA release buffer and elution buffer containing protease K. The final volume of purified DNA was 20 μL.

### Measurement of H3K9me2 levels at the promoter regions of the BDNF gene by real-time PCR

To evaluate the levels of H3K9me2 in the promoter regions of the BDNF gene, the amount of DNA associated with H3K9me2 was measured by quantitative PCR using the CFX96 touch real-time PCR detection system (Bio-Rad). Primers and TaqMan MGB hybridization probes for amplification of promoter regions of the BDNF gene are listed in Table 1 (Fuchikami et al. 2009).

**Table 1:** Primer and probe sequences used for real-time PCR procedure (for quantification of H3K9me2-immunoprecipitated DNA)

Promoter I	Forward Primer	5’-TGATCATCACTCACGACCACG-3’
	Reverse Primer	5’-CAGCCTCTCTGAGCCAGTTACG-3’
	Taq Man MGB Probe	5’-CCAAGGGAGTCACAGTGA-3’
Promoter IV	Forward Primer	5’-TGCAGGGGAATTAGGGATACC-3’
	Reverse Primer	5’-TCTTCGGTTGAGCTTCGATTG-3’
	Taq Man MGB Probe	5’-TTCCGAGGGCTGAA-3’

Minor groove binder (MGB) is a Tm enhancer that increases sequence specificity. FAM (6-carboxyfluorescein) was used as quencher dye. TAMRA (6-carboxytetrame- thylrhodamine) was used as reporter dye

The amount of input DNA was measured simultaneously for normalization of the data. A standard curve was constructed from pooled ChIP samples, and this standard curve was used to measure H3K9me2 levels in unidentified samples. The amounts of DNA associated with H3K9me2 were normalized to the amount of input DNA and calculated as the ratio of target transcript to input DNA.

### Statistical analysis

All values are expressed as mean ± SEM. Experiments containing two groups (Experiments 1–3) were analyzed by an independent t test. To compare BDNF mRNA expression among four groups of rats (Experiment 4), a two-way ANOVA (factors: Stress, Drug) was performed, followed by a post hoc multiple comparisons test. In Experiment 5, the freezing times of the three groups were analyzed using a two-way ANOVA (factors: day, group) for repeated measures (day) followed by a post hoc multiple comparisons test. Significance was determined at *p* < 0.05. R software was used for all statistical analysis.

## Results

### Experiment 1: Effect of SPS on BDNF mRNA levels in the *hippocampus* and in the cerebellum prior to administration of CFC, and before and after extinction training

**Fig. 2:**
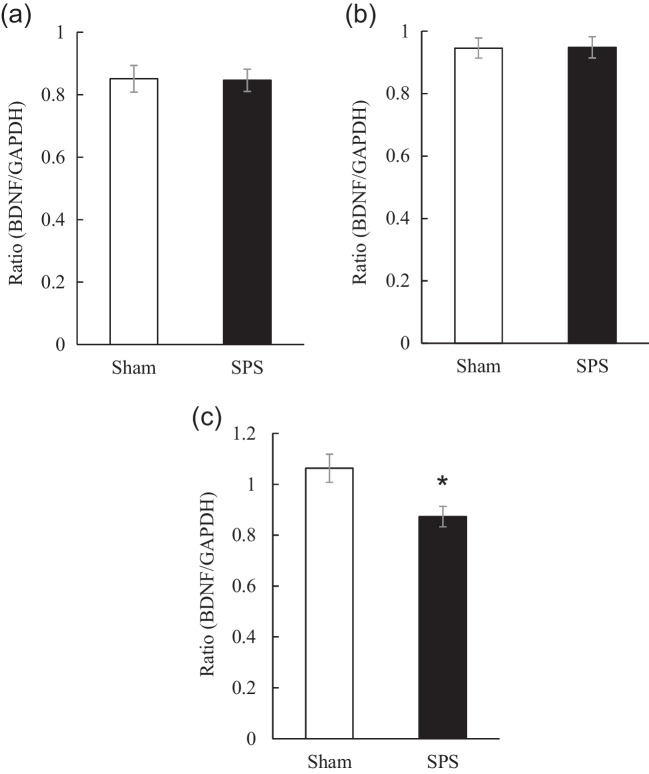
Effect of SPS on hippocampal BDNF mRNA levels (**a**) prior to contextual fear conditioning, (**b**) before extinction training, and (**c**) 2 h after extinction training. Data are expressed as the ratio of BDNF mRNA to GAPDH mRNA (BDNF/GAPDH) and shown as the mean ± SEM (*N* = 8 to 15 rats per group). * *P* < 0.05; independent *t* test

Since the production of BDNF in the rat hippocampus is essential for EFM (Peters et al. 2010), and the decline in BDNF signaling is, at least, associated with EFM impairment in SPS rats (Kataoka et al. 2019), we first examined hippocampal BDNF mRNA levels in SPS rats prior to CFC (time point #1 in Fig. 1), and one day after CFC before and after extinction training (time points #2 and #3 in Fig. 1). While hippocampal BDNF mRNA levels neither prior to CFC [t(24) = 0.09, df = 24, *P* = 0.93] (Fig. 2a) nor before extinction training [t(28) = -0.05, df = 28, *P* = 0.96] (Fig. 2b) significantly differed between SPS and sham rats, the levels of hippocampal BDNF mRNA were significantly decreased at 2 h after extinction training in SPS rats compared to sham rats [t(14) = 2.77, df = 14, *P* < 0.05] (Fig. 2c). On the other hand, no significant differences in the levels of BDNF mRNA in the cerebellum between SPS and sham rats were seen prior to CFC [t(10) = 0.016, df = 10, *P* = 0.99] (Supplemental Fig. 1a), and before extinction training [t(10) = -1.28, df = 10, *P* = 0.23] (Supplemental Fig. 1b), and after extinction training [t(10) = -1.03, df = 10, *P* = 0.33] (Supplemental Fig. 1c).

### Experiment 2: Effect of SPS on global H3K9me2 levels in the *hippocampus* and in the cerebellum

**Fig. 3:**
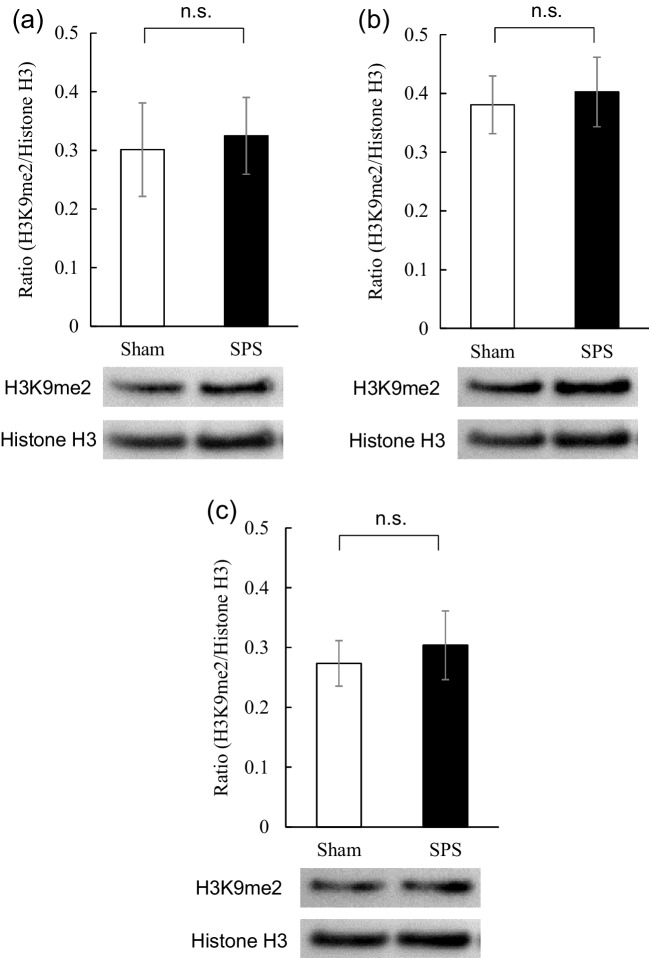
Effect of SPS on global H3K9me2 levels in rat hippocampus (**a**) prior to contextual fear conditioning, (**b**) before extinction training, and (**c**) 2 h after extinction training. (Top) quantification of global H3K9me2 levels (Bottom) representative western blot for H3K9me2 and Histone H3. Data are expressed as the ratio of H3K9me2 to histone H3 (H3K9me2/histone H3) and shown as the mean ± SEM (*N* = 7 to 8 rats per group)

Since the levels of BDNF mRNA were significantly decreased in SPS rats 2 h after extinction training, we next investigated the effect of SPS on global H3K9me2 levels in the hippocampus by Western blotting. No significant differences were found in these levels between SPS and sham rats prior to CFC (time point #1 in Fig. 1 [t(14) = -0.23, df = 14, *P* = 0.82] and Fig. 3a), before extinction training (time point #2 in Fig. 1 [t(13) = -0.29, df = 13, *P* = 0.78] and Fig. 3b), or after extinction training (time point #3 in Fig. 1 [t(14) = -0.44, df = 14, *P* = 0.67] and Fig. 3c). On the other hand, no significant change in the levels of H3K9me2 in the cerebellum between SPS and sham rats were seen prior to CFC [t(10) = -0.29, df = 10, *P* = 0.78] (Supplemental Fig. 2a), and before extinction training [t(10) = -0.24, df = 10, *P* = 0.81] (Supplemental Fig. 2b), and after extinction training [t(10) = 0.18, df = 10, *P* = 0.86] (Supplemental Fig. 2c).

### Experiment 3: Effect of SPS on H3K9me2 levels at the promoter regions of the BDNF gene in the rat *hippocampus*

**Fig. 4:**
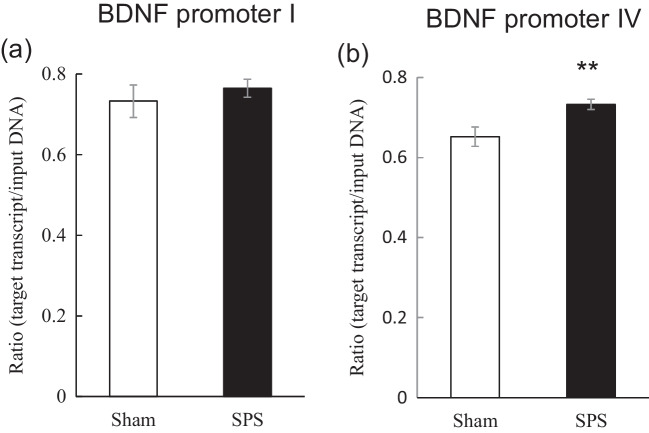
Effect of SPS on H3K9me2 levels at the promoter regions of the BDNF gene in the rat hippocampus prior to the extinction training. Data are expressed as the ratio of the target sequence (BDNF promoter I or IV) to that of input DNA (target sequence/input DNA) and shown as the mean ± SEM (*N* = 10 rats per group). ** *P* < 0.01; independent *t* test

To elucidate the mechanism of decrease in the hippocampal BDNF mRNA levels of SPS rats 2 h after the extinction training, we investigated the influence of SPS on H3K9me2 levels in the promoter regions of the BDNF gene prior to extinction training (time point #2 in Fig. 1). Whereas H3K9me2 level in promoter I did not significantly differ between SPS and sham rats [t(18) = -0.69, df = 18, *P* = 0.50] (Fig. 4a), the level in promoter IV was significantly higher in SPS than sham rats [t(18) = -2.95, df = 18, *P* < 0.01] (Fig. 4b).

### Experiment 4: Effect of intra hippocampal administration of BIX01294 on SPS-induced decrease in the hippocampal BDNF mRNA levels

**Fig. 5:**
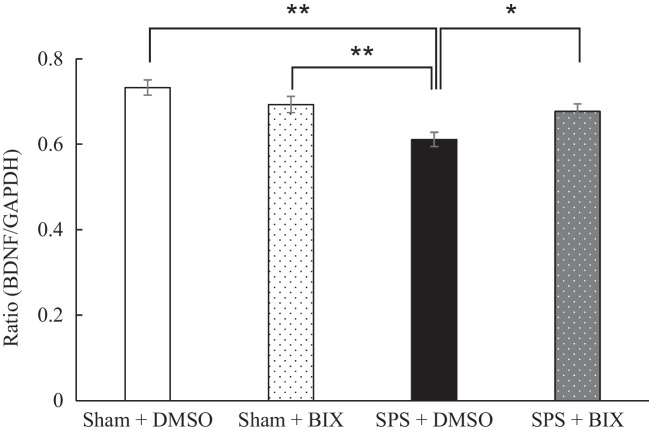
Effect of intra-hippocampal administration of BIX01294 on SPS-induced decrease in the hippocampal BDNF mRNA levels. Data are expressed as the ratio of the BDNF mRNA to GAPDH mRNA (BDNF/GAPDH) and shown as the mean ± SEM (*N* = 10 to 11 rats per group). * *P* < 0.05, ** *P* < 0.01; one-way ANOVA with Tukey post-hoc analysis

We next examined the effect of an HMT G9a inhibitor (BIX01294) on the levels of BDNF mRNA in the hippocampus 2 h after extinction training. Two-way ANOVA demonstrated a significant main effect of stress [F(1,42) = 20.05, *P* < 0.01], but not of drug [F(1,42) = 0.43, *P* = 0.514]. There was a significant stress x drug interaction [F(1.42) = 12.20, *P* = 0.012]. Post-hoc analysis revealed that BDNF mRNA levels in the hippocampus of the SPS + DMSO group were significantly lower than those of the Sham + DMSO (*P* < 0.01) and SPS + BIX01294 (*P* = 0.021), and Sham + BIX01294 groups (*P* < 0.01), and the levels of BDNF mRNA did not significantly differ among the Sham + DMSO and Sham + BIX01294 and SPS + BIX01294 groups (Fig. 5).

### Experiment 5: Effect of intra hippocampal administration of BIX01294 on impaired EFM in SPS rats

**Fig. 6:**
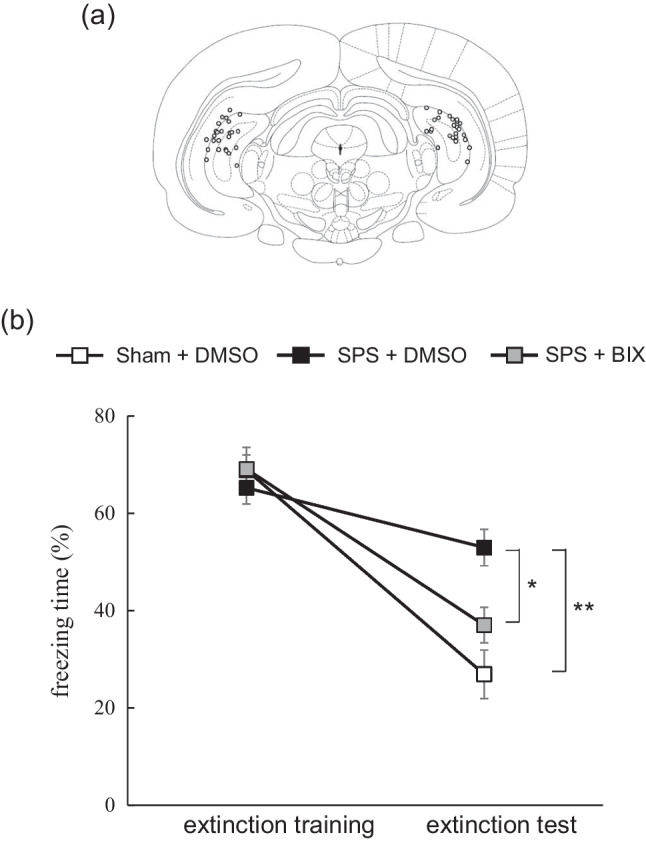
Effect of BIX01294 administration on impaired fear memory extinction in SPS rats. **a** Schematic drawing of coronal section of the rat ventral hippocampus showing locations of bilateral infusion sites according to the brain atlas of (Paxinos and Watson 1998). **b** Result of behavioral test. Data are expressed as the mean percentage of freezing behavior during extinction training and testing ± SEM (*N* = 15 to 25 rats per group). * *P* < 0.05, ** *P* < 0.01; two-way ANOVA with Tukey post-hoc analysis

Regarding the effect of BIX01294 injection into the hippocampus, two-way ANOVA demonstrated significant main effects of day [F(1,59) = 82.78, *P* < 0.01], group [F(2,59) = 3.88, *P* < 0.05], and a significant day x group interaction [F(2,59) = 7.12, *P* < 0.01]. Post hoc analysis found no significant difference in freezing time during extinction training among the 3 groups. During extinction test sessions, freezing time was significantly longer in the SPS + DMSO group than the Sham + DMSO group (*P* < 0.01) and SPS + BIX01294 group (*P* < 0.05), and did not significantly differ between the Sham + DMSO and SPS + BIX01294 groups (Fig. 6b).

## Discussion

EFM impairment is closely linked to intrusion symptoms in patients with PTSD, and the alleviation of intrusion symptoms is necessary for the efficacious treatment of PTSD. Although the role of BDNF in EFM remains largely unknown, several studies have demonstrated the involvement of BDNF in the development of EFM. For example, using a CFC paradigm in rats, Peters et al. (2010) demonstrated that intra-hippocampal microinfusion of BDNF evoked EFM. Rosas-Vidal and his colleagues (Rosas-Vidal et al. 2014) reported that extinction training induces the initial increase in hippocampal BDNF protein. Without the measurement of EFM, several studies demonstrated that SPS treatment decreased BDNF levels in the hippocampus (Lee et al. 2016; Mirjalili et al. 2022; Shafia et al. 2017). In addition, we have previously reported that SPS rats showed enhanced fear memory consolidation, accompanied by increased levels of BDNF transcripts and protein in the hippocampus (Takei et al. 2011). From these reports, it is suggested that while the reduction of BDNF levels in the hippocampus may play a pivotal role in the impaired EFM, increase in BDNF levels may be associated with enhanced fear memory in PTSD, though no relationship between hippocampal BDNF levels and the impaired fear memory has been demonstrated in humans. In this context, we examined changes in hippocampal BDNF mRNA levels in SPS rats subjected to CFC at various time points, and subsequently evaluated the involvement of H3K9me2 in the transcription of the BDNF gene in the hippocampus. As a result, hippocampal levels of BDNF mRNA 2 h after extinction training were found to be significantly lower in SPS rats exhibiting EFM impairment than in sham rats.

In addition to the significantly reduced hippocampal levels of BDNF mRNA, we found significantly increased binding of H3K9me2 to the promoter IV of the BDNF gene in the hippocampus prior to the extinction training in SPS rats without any change in the global level of H3K9me2. Recently, one of the epigenetic modifications, the methylation status of H3K9, was shown to play a critical role in the transcriptional control of the genes in the brain (Fuks 2005; Li et al. 2007). For example, H3K9me2 was reported to correlate with transcriptional silencing while H3K4me3 was shown to be involved in transcriptional activation (Santos-Rosa et al. 2002; Schneider et al. 2004). In this context, to elucidate the involvement of H3K9me2 in reduced hippocampal levels of BDNF mRNA in SPS rats, we measured levels of global H3K9me2 by Western blotting, and the binding of H3K9me2 to the promoters of the BDNF gene by ChIP assay with quantitative real-time PCR. Although no significant difference was found in the global level of H3K9me2, there was significant enhancement of the level of H3K9me2 binding to promoter IV, but not promoter I, in the hippocampus of SPS rats 2 h after extinction training. In line with the effect of H3K9me2 on the transcription of the BDNF gene in the hippocampus, Gupta-Agrawal and colleagues used the ChIP assay with quantitative real-time PCR to show the increase in H3K9me2 level in the promoter of exon IV of the BDNF gene with decreased levels of exon IV mRNA in the rat entorhinal cortex and hippocampus during memory consolidation. Also using ChIP assay with quantitative real-time PCR (Gupta-Agarwal et al. 2012), Tian and coworkers (Tian et al. 2009) demonstrated that NMDA receptor activation increased BDNF exon IV mRNA and decreased H3K9me2 binding to the promoter of exon IV in hippocampal neurons. Despite the direct involvement of H3K9me2 in the BDNF gene expression, (Boulle et al. 2014) reported that levels of H3K9me2 decreased and levels of hippocampal BDNF mRNA increased in mice exposed to unpredictable socio-environmental stressors, suggesting the importance of H3K9me2 to BDNF gene transcription. In addition, unlike H3K9 methylation, methylation of CpGs upstream of exon IV of the BDNF gene plays a pivotal role in the transcription of the BDNF gene (Chen et al. 2003; Yasuda et al. 2009). Collectively, the data suggest the possibility that increased H3K9me2 in the promoter of exon IV may downregulate BDNF mRNA levels in the hippocampus of SPS rats 2 h after extinction training.

Since one of the treatment-resistant symptoms of PTSD is flashbacks, it is noteworthy that administration of BIX01294 16 h before extinction training markedly reduced the freezing time during the extinction test session in SPS rats. Following the procedure of Gupta et al. (Gupta-Agarwal et al. 2012), we injected 45 µM of BIX01294 into the hippocampus in this study. Although the binding levels of H3K9me2 to the promoter IV of the BDNF gene were not measured after BIX01294 intra-hippocampal administration, they should have been decreased in the hippocampus of SPS rats. In fact, no significant difference was found in the hippocampal BDNF mRNA levels between BIX01294-treated SPS rats and sham rats 2 h after extinction training. As mentioned above, the hippocampal-prefrontal BDNF plays an important role in the induction of EFM (Peters et al. 2010; Rosas-Vidal et al. 2014). In addition, rapid and selective induction of BDNF expression in the hippocampus was reported to play an important role in the consolidation of fear memory (Hall et al. 2000; Lubin et al. 2008). In particular, Lubin et al. (2008) demonstrated that transcription of exon-IV of the BDNF gene was upregulated in the rat hippocampus CA1 region 2 h after context exposure and footshock. Considered together with these findings, our findings suggest that induction of the BDNF gene in the hippocampus of SPS rats 2 h after extinction training is lower because contextual consolidation is weak in the absence of footshock, and lower induction may be involved in EFM impairment via increased binding of H3K9me2 to the promoter of exon IV. Thus, it is plausible that the amelioration by BIX01294 of reduced transcription of the BDNF gene 2 h after extinction training may contribute to the recovery from EFM impairment in SPS rats. Because of faster effects of BIX01294 administration on BDNF transcription in the hippocampus (Gupta-Agarwal et al. 2012) and on long-term potentiation in the hippocampus (Sharma et al. 2017), further experiments examining the most effective timepoint of administration to alleviate the impaired EFM of SPS rats are needed.

In summary, the results of the present study demonstrate the involvement of hippocampal H3K9me2 in the development of intrusion symptoms of PTSD and suggest the possibility that the inhibition of H3K9me2 may provide a new approach to PTSD treatment. However, our results also indicate that the transcriptional regulation of the BDNF gene mediated by the status of H3K9me2 and the influence of BIX01294 on the acetylation of H3 (Gupta et al. 2010; Lubin et al. 2008). Collectively, the evidence indicates that comprehensive studies looking at the methylation and acetylation of histone simultaneously in the hippocampus of SPS rats will be needed to reveal the precise epigenetic mechanism and develop newer treatments for PTSD.

## Supplementary Information

Below is the link to the electronic supplementary material.

Supplementary file1 (DOCX 270 KB)
