# Supplementary material for: Involvement of dysregulated hippocampal histone H3K9 methylation at the promoter of the BDNF gene in impaired memory extinction
Source: Psychopharmacology (Berl). 2024 Jun 28;241(11):2363–74. doi: 10.1007/s00213-024-06640-7 (PMC11513706; doi:10.1007/s00213-024-06640-7)
Supplement: Supplementary file 1 — Supplementary file1 (DOCX 270 KB) [file 213_2024_6640_MOESM1_ESM.docx]

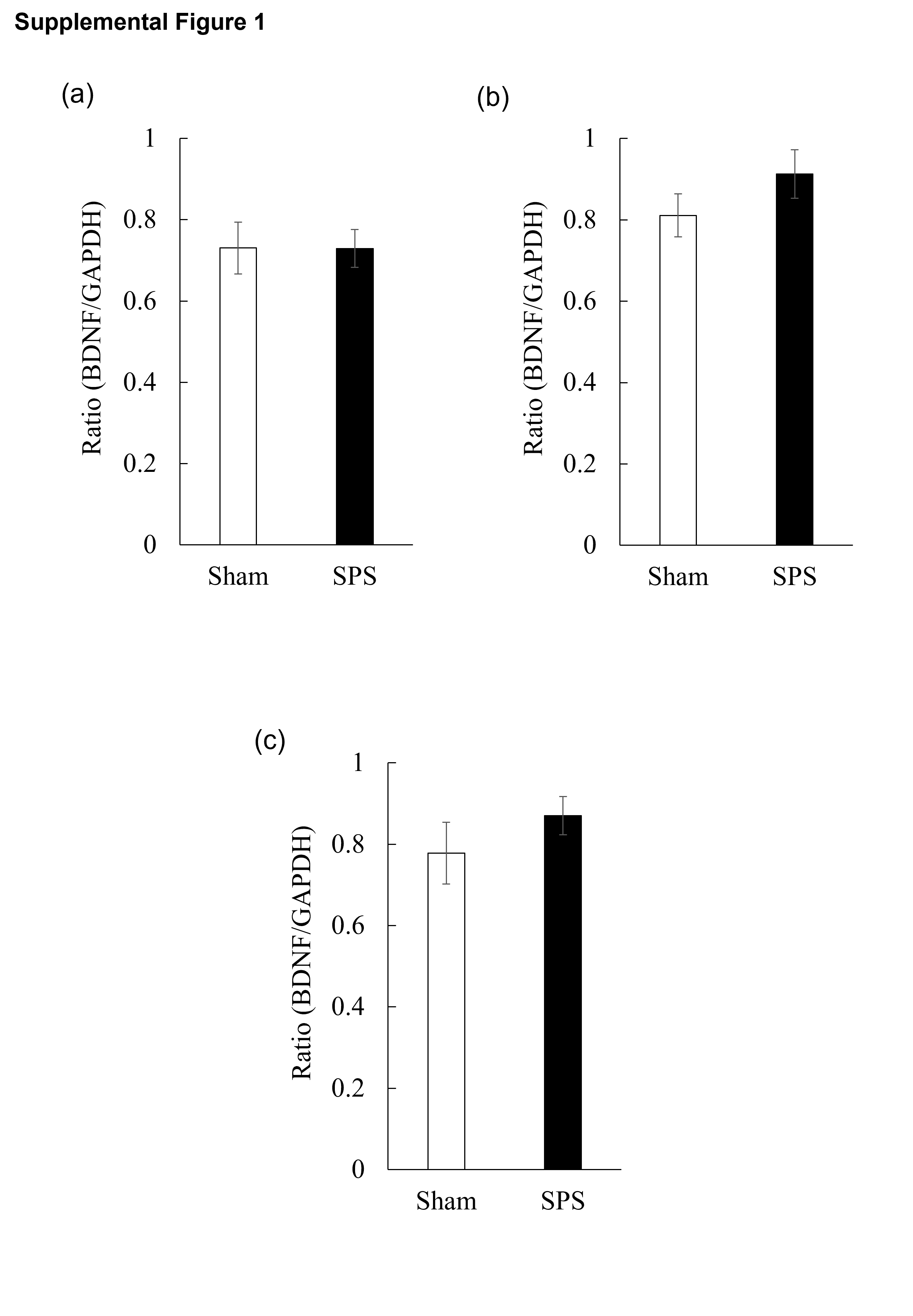


**Suppl. Fig 1. Effect of SPS on Cerebellar BDNF mRNA levels (a) prior to contextual fear conditioning, (b) before extinction training, and (c) 2 hours after extinction training.**

Data are expressed as the ratio of BDNF mRNA to GAPDH mRNA (BDNF/GAPDH) and shown as the mean ± SEM (N = 6 rats per group).


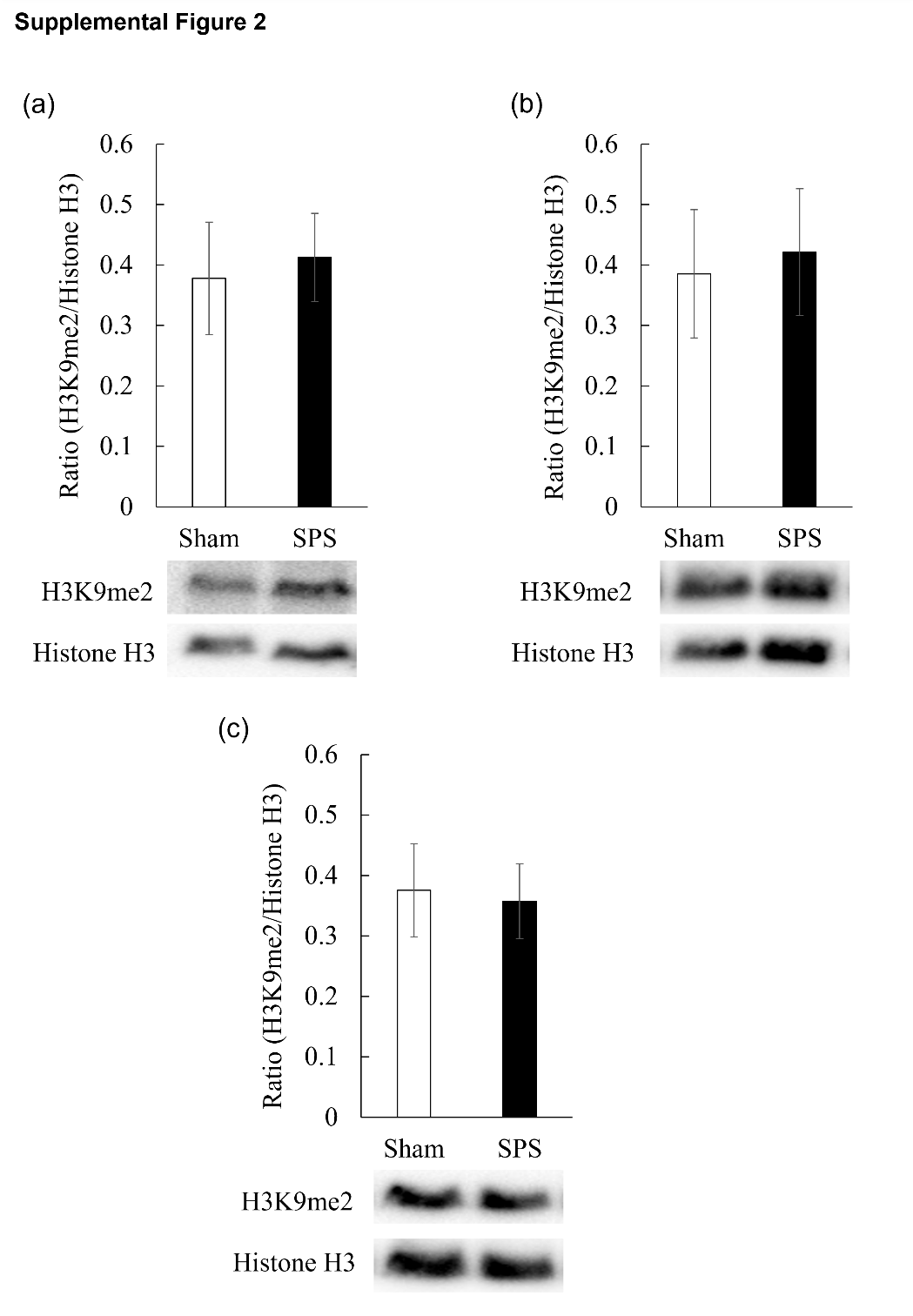


**Suppl. Fig 2. Effect of SPS on global H3K9me2 levels in rat cerebellum (a) prior to contextual fear conditioning, (b) before extinction training, and (c) 2 hours after extinction training.**

(Top) quantification of global H3K9me2 levels（Bottom）representative western blot for H3K9me2 and Histone H3

Data are expressed as the ratio of H3K9me2 to histone H3 (H3K9me2/histone H3) and shown as the mean ± SEM (N = 6 rats per group).
